# Mass Spectrometry-based Solutions for Single-cell Proteomics

**DOI:** 10.1093/gpbjnl/qzaf012

**Published:** 2025-02-22

**Authors:** Siqi Li, Shuwei Li, Siqi Liu, Yan Ren

**Affiliations:** HIM-BGI Omics Center, Hangzhou Institute of Medicine (HIM), Chinese Academy of Sciences, Hangzhou 310022, China; BGI-Shenzhen, Shenzhen 518083, China; Nanjing Apollomics Biotech Inc., Nanjing 210033, China; China Pharmaceutical University, Nanjing 210009, China; HIM-BGI Omics Center, Hangzhou Institute of Medicine (HIM), Chinese Academy of Sciences, Hangzhou 310022, China; BGI-Shenzhen, Shenzhen 518083, China; HIM-BGI Omics Center, Hangzhou Institute of Medicine (HIM), Chinese Academy of Sciences, Hangzhou 310022, China; Experiment Center for Science and Technology, Shanghai University of Traditional Chinese Medicine, Shanghai 201203, China

**Keywords:** Mass spectrometry-based single-cell proteomics, Single-cell isolation, Sample preparation, Liquid chromatography separation, Data acquisition and analysis

## Abstract

Mass spectrometry-based single-cell proteomics (MS-SCP) is attracting tremendous attention because it is now technically feasible to quantify thousands of proteins in minute samples. Since protein amplification is still not possible, technological improvements in MS-SCP focus on minimizing sample loss while increasing throughput, resolution, and sensitivity, as well as achieving measurement depth, accuracy, and stability comparable to bulk samples. Major advances in MS-SCP have facilitated its application in biological and even medical research. Here, we review the key advancements in MS-SCP technology and discuss the strategies of the typical proteomics workflow to improve MS-SCP analysis from single-cell isolation, sample preparation, and liquid chromatography separation to MS data acquisition and analysis. The review will provide an overall understanding of the development and applications of MS-SCP and inspire more novel ideas regarding the innovation of MS-SCP technology.

## Graphical abstract

**Figure:**
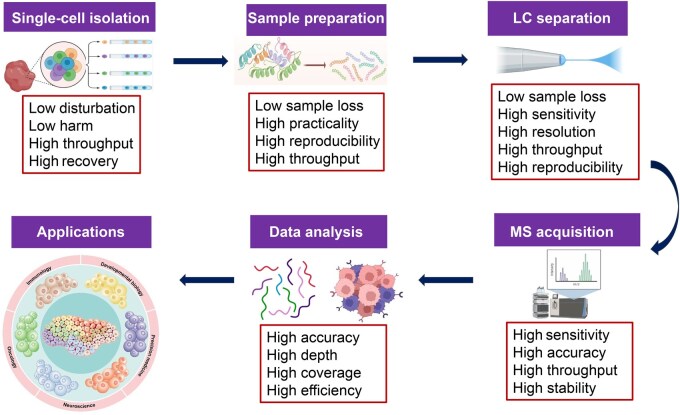

## Introduction

Cell diversity is essential to all life systems, particularly at higher levels of organization. Understanding the variety of cells under different conditions is beneficial for controlling the development of life systems and promoting progress in the medical sciences, including the diagnosis, treatment, and prognosis of disease [1]. Single-cell omics, which refers to large-scale profiling of gene status and gene expression or metabolites in individual cells, could provide deeper insights into cell diversity and its relevant biological significance. The single-cell RNA sequencing (scRNA-seq) technique has made significant advancements since it was first introduced in 2009 [2]. Recent scRNA-seq technologies like GEM-X Flex (10X Genomics) can profile up to 2.56 million cells per run (https://www.10xgenomics.com/platforms/chromium/technology). The massive data generated from single-cell genomic and transcriptomic studies have not only revolutionized our understanding of cellular heterogeneity, cell population characterization, and immune diversity, but also demonstrated a powerful potential in fields such as cancer medicine, reproductive medicine, regenerative medicine, and personalized medicine [3]. However, it is already known that RNA levels may not reflect protein levels well due to post-transcriptional regulation. Specht et al. stated that Single-Cell ProtEomics (SCoPE2) method measured 20-fold more protein copies than RNA copies by 10X Genomics (scRNA-seq) per gene [4]. The summary on the comparison of single-cell transcriptomics and single-cell proteomics (SCP) is listed in **Table 1**.

**Table 1:** Comparison between single-cell transcriptomics and SCP

	Single-cell transcriptomics	SCP
Year initiated	2009	2018
Representative methods	sci-Plex [5], FLASH-seq [6], GEM-X*	SCoPE2 [4], nanoPOTS [7], proteoCHIP [8]
Advantages	1. High throughput	1. Proteins are functional molecules
	2. Relatively developed techniques	2. Direct correlation with biological activities
	3. Relatively comprehensive coverage	3. Capture proteins’ PTMs, interactions, and subcellular localization
Disadvantages	1. Transcripts are not functional molecules in cells	1. Low throughput
	2. Limited correlation with phenotypes	2. Limited coverage
	3. Poor proxies to proteomics	3. Low sensitivity to low-abundance proteins

*Note*: * GEM-X, https://www.10xgenomics.com/platforms/chromium/technology. SCP, single-cell proteomics; SCoPE2, Single-Cell ProtEomics; nanoPOTS, nanodroplet processing in one pot for trace samples; PTM, post-translational modification.

Since proteomics was initiated in the 1990’s, its important role in directly revealing cell functions has been highlighted due to the fact that life activity mainly relies on protein expression, modification, and interaction networks. With developments in omics, proteomics has extended to the single-cell level, while SCP technology has attracted great attention for its ability to elucidate cell functions. Compared with well-established single-cell genomics and transcriptomics technologies, SCP is still a fast-developing field [9]. Currently, the primary task of SCP technology is to identify and quantify as many proteins as possible at the single-cell level within an acceptable timeframe. It is generally recognized that the lack of protein amplification methods is a barrier to identifying a broad dynamic range of proteins in cells and tissues. Although the expressed genes in cells vary from tens to tens of millions, the current technology could identify a maximum of 12,000 proteins in bulk samples [10,11]. Therefore, the challenge of SCP technology is how to improve the detection sensitivity of proteins at the single-cell level.

There are multiple techniques involved in the development of SCP. Based on the principle of protein detection, the current SCP technologies are generally divided into two categories: antibody-based SCP and mass spectrometry (MS)-based SCP (MS-SCP). A main technological consideration of antibody-based SCP is that cell features are recognized by antibodies, while antibodies conjugated with signals are indicative for single cells. To capture antigen signals in single cells, two methods are commonly employed: (1) the single-cell barcode chip that uses an array of immobilized antibody strips to measure single-cell proteins, and (2) the proximity ligation assay (PLA) that uses pairs of DNA-functionalized antibodies to detect single-cell proteins [12]. In antibody-based SCP, fluorescence-activated cell sorting (FACS) and immunofluorescence microscopy (IFM) are common instruments for separating single cells and exploring protein localization [13]. Cytometry by time of flight (CyTOF), which combines the principles of flow cytometry and MS, quantifies approximately 40 targets with metal-coded antibodies in single cells with higher throughput and a lower limit by overlapping spectra instead of fluorophore-based tagging [14–16]. However, the antibody-based SCP technology is impeded by two challenges: (1) how to reduce noise interference to detect proteins at the low attomolar range in single cells, and (2) how to expand antibodies with minimal nonspecific binding and ultra-low dissociation constants (Kd). MS-SCP is an unsupervised approach for comprehensively analyzing proteomes at the single-cell level through sensitive and fast scanning of tandem MS (MS/MS) signals from peptides and robust computational algorithms for transforming MS raw data into accurate protein quantification. Although there are still many technological obstacles in MS-SCP, it is generally accepted that this method enables large-scale protein measurement down to the single-cell level. Each mammalian cell contains only 100–200 pg of proteins, which cannot be amplified. Compared with the proteomic analysis of bulk samples, MS-SCP focuses on the modification of typical proteomics workflow to increase its sensitivity, depth, and throughput for trace-level protein identification and quantification. A series of solutions have been adopted to promote MS-SCP analysis, and have been applied to reveal the diversity of cell populations and functions in different biological events. In this review, we discuss the strategies in each step of the typical proteomics workflow to improve MS-SCP analysis, along with its technological advancements, including feasibility, limitations, and potential development.

## MS-SCP

MS can accurately measure thousands of proteins and their post-translational modifications (PTMs) in a single run, which provides more comprehensive information at the proteomic level. One of the most common workflows is the bottom-up strategy, in which proteins are extracted from biological samples, digested with site-specific proteases (*e.g.*, trypsin), and then analyzed by MS/MS. Peptides are centrifuged and desalted to remove debris and MS-incompatible chemicals before they are subjected to liquid chromatography (LC) for separation. During the separation, eluted peptides from LC are immediately and continuously transported into MS, where they are ionized, acquired in real-time, and recorded as two types of MS spectra [17]. MS1 spectra contain information about precursors (intact peptides), and MS2 (MS/MS) spectra record information about product ions (peptide fragments). Collectively, MS1 and MS2 spectra can be used to identify specific peptide sequences and quantify their abundance, and the data from peptides derived from the same protein are combined to infer the identity and abundance of the parent protein [18,19].

**Figure 1:**
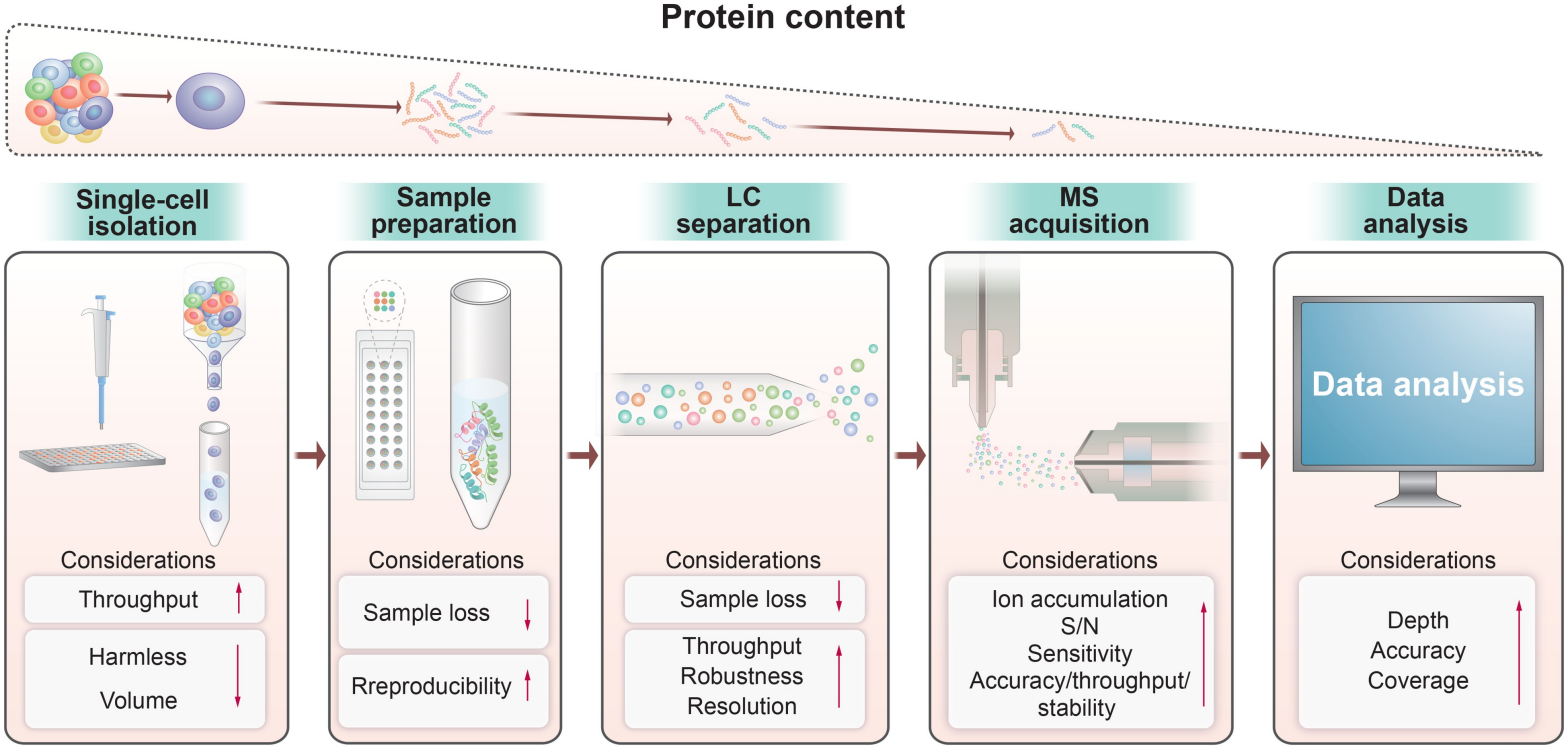
Impacts on improving MS-SCP technologies following each step of the typical proteomics workflow MS, mass spectrometry; MS-SCP, mass spectrometry-based single-cell proteomics; LC, liquid chromatography; S/N, signal-to-noise.

Although the required input material for MS-based proteomics research has dropped from micrograms to nanograms, the MS approach still faces the most common challenge in the SCP field, *i.e.*, the extremely trace amounts of materials that contain thousands of proteins with a wide dynamic range. Single cells with higher protein yields (such as larger neurons, human oocytes, or *Xenopus* embryos) were the initial choices in early-stage MS-SCP analysis, due to their higher protein content than other single cells [20–22]. With numerous efforts in cell isolation, sample preparation, peptide separation, data acquisition, and bioinformatic processing (**Figure 1**), MS-SCP has now expanded to cancer cells, stem cells, cardiomyocytes, and monocytes, achieving a depth of 1000–5000 proteins [8,23–27].

**Figure 2:**
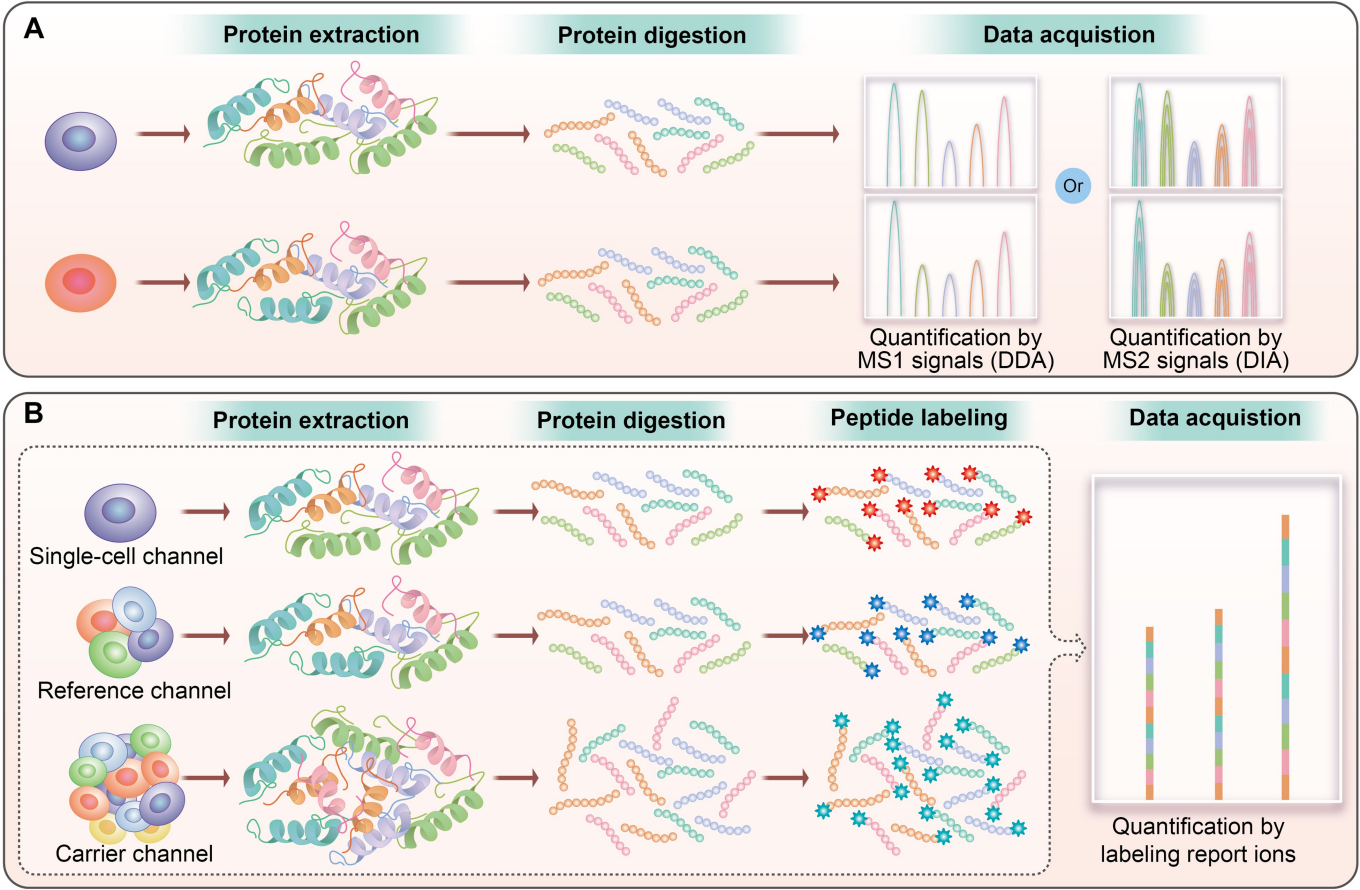
Two types of quantification strategies on MS-SCP **A**. Label-free quantification workflow for MS-SCP. **B**. Labeled quantification workflow for MS-SCP. DDA, data-dependent acquisition; DIA, data-independent acquisition.

Following the progress of traditional proteomics, MS-SCP technology has proceeded to labeled and label-free quantification approaches, each with both advantages and challenges (**Figure 2**). The labeled methods for MS-SCP provide higher throughput, since more than 10 single cells can be quantified in a single run [4]. However, a common channel utilized as a bridge in each run will be necessary for the quantification of numerous single cells. To improve protein identification from single cells, a carrier channel with hundreds of cells (approximately tens of nanograms of proteins) is used for individually labeling and then mixed with different channels of labeled single cells for MS analysis [28]. It limits the quantification accuracy due to the adverse impact of carrier proteins on data quality [29]. The single-cell channels are individually labeled during sample preparation, and the mixing of these channels increases the operation steps and the variation of quantification. Due to the ratio compression in labeled quantification methods, the diversity of cell populations inferred according to the differential abundance in single cells might be diluted [29]. Because labeling reagents are employed, the cost of labeled quantification of single cells is definitely higher than that of label-free quantification during sample preparation. In contrast, the label-free method for MS-SCP offers significantly lower throughput because the single-cell samples are separately analyzed [26]. The benefits of the label-free method include simpler sample preparation, greater protein coverage, higher quantification accuracy, and lower costs of sample preparation.

For establishing MS-SCP methods (labeled or label-free), the major considerations are to prevent sample loss and increase sensitivity during each step of the whole proteomics workflow (Figure 1). Strategies include minimizing operation steps, reducing processing volumes, and employing booster samples, nanodroplets, oil–air–droplet (OAD) systems, coated peptides, or mass spectrometers with higher detection sensitivity [26,28,30–35]. To reduce losses from individual single cells, a carrier channel with 200 cells is often employed in labeled quantification, since most of the peptides lost after mixing them with single cells due to surface absorption originate from the carrier cells, thereby assisting in the identification of single-cell proteins owning to their higher protein copies [28]. To highlight the efforts to accelerate MS-SCP analysis in different trials, in this review, we elucidate them in detail following the proteomics workflow. This review will provide an overall understanding of the development and applications of MS-SCP and inspire novel ideas on the innovation of MS-SCP technology.

### Single-cell isolation

SCP studies usually start by isolating single cells from bulk samples. This approach can be challenging for adherent cell cultures and tissues, which requires vigorous detachment or dissociation procedures, including enzyme digestion and scraping [36]. Dissociated cells should be thoroughly washed to eliminate contaminants incompatible with the downstream analysis. FACS has been widely used to sort and collect individual cells from single-cell suspensions (**Table 2**). Despite its operational simplicity, high accuracy, and high throughput, FACS requires a large initial sample volume due to its long tubing system [37]. Additionally, the high pressure and high vibration frequency of the sorting system decrease the cell viability and survival rate. Recently, an imaging-based single-cell isolation device, CellenONE, was adopted to isolate and collect single cells, followed by sequential proteomic sample preparation steps [38]. Compared with FACS, CellenONE requires significant fewer starting cells for single-cell sorting and achieves higher post-sorting cell viability. There are alternative commercial devices for single-cell sorting, but their costs are much higher because expensive disposable chips or cassettes are needed. The Tecan Uno Single Cell Dispenser (formerly HP D100 Single Cell Dispenser), coupled with 384-well polymerase chain reaction (PCR) plates, is an affordable alternative option. It integrates cell isolation and sample preparation but cannot isolate specific cell subpopulations from mixed samples like FACS or CellenONE [39].

**Table 2:** Overview of recent advances in the development of MS-SCP technology

Cell type	Cell isolation	Sample preparation	LC-MS	Acquisition mode	Raw data searching	Protein count	Ref.
HeLa	Microscope, self-aligning monolithic device	OAD protocol, on chip, 100 nl volume	50 μm × 15 cm C18 column, Orbitrap Elite MS	LFQ-DDA	IP2 platform	51	[34]
HeLa	Microscope, syringe pump, and capillary	iPAD-1 protocol, in capillary, 2 nl volume	22 μm × 3 cm C18 column, Orbitrap Fusion Tribrid MS	LFQ-DDA	MaxQuant	328	[40]
HeLa	FACS	nanoPOTS protocol, on chip, 200 nl volume	Narrow-bore column (30 μm × 50 cm), Orbitrap Fusion Lumos MS	LFQ-DDA	MaxQuant	670	[41]
HeLa	Nanopipetting robot	nanoPOTS protocol, on chip, 200 nl volume	Narrow-bore column (20 μm × 60 cm), Orbitrap Eclipse Tribrid MS	LFQ-DDA	MaxQuant	874	[42]
HeLa, human neuron	Nanopipetting robot	nanoPOTS protocol, on chip, 200 nl volume	Narrow-bore column (20 μm × 50 cm), Orbitrap Eclipse Tribrid MS with FAIMS Pro	LFQ-DDA	Proteome Discoverer, MaxQuant	1056 in HeLa, 1085 in neuron	[30]
HeLa, lymphocyte	FACS	autoPOTS (automated version of nanoPOTS), in 384-well plate, 4 μl volume	30 µm × 45 cm C18 column, Orbitrap Exploris 480 MS	LFQ-DDA	MaxQuant	301 in HeLa	[43]
HeLa, K562, Jurkat	CellenONE	nanoPOTS protocol, on chip, 200 nl volume	Two-column LC system, 50 μm × 10 cm C18 column, Orbitrap Exploris 480 MS	LFQ-DDA	Proteome Discoverer	∼ 1000 in HeLa	[44]
HeLa, human CD34^+^ cell	FACS	Mad-CASP protocol, in tube, 8 μl volume	75 µm × 30 cm C18 column, Fusion Lumos Tribrid MS	LFQ-DDA	MaxQuant	1240 in HeLa, ∼ 1000 in CD34^+^ cell	[33]
HeLa	CellenONE X1	One-step preparation on polypropylene chip, 200 nl volume	30 µm × 30 cm C18 column, Orbitrap Eclipse MS	LFQ-DDA	FragPipe	∼ 2400	[32]
HeLa	HP D100 Single Cell Dispenser	One-step preparation in 384-well PCR plate, 500 nl volume	30 µm × 30 cm C18 column or 50 µm × 15 cm C18 column, Orbitrap Exploris 480 MS	LFQ-DDA	Proteome Discoverer	2572 with 30 μm i.d. column, 1668 with 50 μm i.d. column	[39]
Jurkat, U-937	FACS	SCoPE-MS, in tube, ∼ 4.5 μl volume	75 μm × 20 cm C18 column, LTQ Orbitrap Elite MS	Multiplexed DDA	MaxQuant	767 in 24 single cells	[28]
Monocyte, macrophage	FACS	SCoPE2 + mPOP protocol, in 384-well plate, 1 μl volume	75 μm × 25 cm C18 column, Q-Exactive MS	Multiplexed DDA	MaxQuant	3042 in 1490 single cells	[4]
C10, Raw264.7, SVEC	CellenONE	Nested nanoPOTS (N2) chip, 30 nl volume	100 μm × 4 cm C18 column, Orbitrap Eclipse Tribrid MS	Multiplexed DDA	MaxQuant	∼ 1700 in 9 single cells	[7]
OCI-AML8227	FACS	In 384-well plate, ∼ 3 μl volume	15 cm C18 column, Orbitrap Exploris 480 MS with FAIMS Pro	Multiplexed DDA	Proteome Discoverer	987	[25]
HeLa, S2, HEK-293T	CellenONE	CellenONE + proteoCHIP system, ∼ 200 nl volume	75 μm × 15 cm C18 column, Orbitrap Exploris 480 MS with FAIMS	Multiplexed DDA	CHIMERYS in Proteome Discoverer	∼ 2000 in 10 single cells	[45]
HeLa	FACS	In 384-well plate, concentrated into 20 nl in StageTip	Evosep One LC, 75 μm × 15 cm C18 column, timsTOF Pro MS	diaPASEF	DIA-NN	∼ 1000 to ∼ 2000	[24]
PC-9, B-CLL, MEC-1	iProChip device	In iProChip device, 78.5 nl volume	75 μm × 25 cm C18 column, Orbitrap Eclipse MS	DIA	Spectronaut	∼ 1500	[31]
HeLa, A549, U2OS	Probe-based microfluidic liquid handling robot	PiSPA workflow, in insert tube of LC autosampler, nanoliter-scale volume	Unknown LC column, timsTOF Pro MS	DIA	DIA-NN	3049	[26]
HEK293, HeLa	FACS	In 384-well plate, 1 μl volume	75 μm × 15 cm C18 column, orbitrap Eclipse Tribrid MS with FAIMS Pro	DIA	Spectronaut	∼ 2000	[46]
HeLa, K563	CellenONE	In 384-well plate, 1 μl volume	5.5 cm μPAC column, Orbitrap ExplorisTM 480 MS with FAIMS Pro	LFQ-DDA, DIA	Spectronaut for DIA, Proteome Discoverer	1790 in DDA, 2200 in DIA	[35]
HeLa	CellenONE	proteoCHIP EVO 96, 300 nl reaction volume	Evosep One LC, Aurora Elite TS columns, Orbitrap Astral MS	nDIA	Spectronaut	∼ 5000	[27]
HEK-293T	CellenONE	proteoCHIP EVO 96, 300 nl reaction volume	Aurora Elite or Rapid columns, timsTOF Ultra MS	DIA	DIA-NN	4000	[8]

*Note*: MS, mass spectrometry; LC, liquid chromatography; i.d., internal diameter; DDA, data-dependent acquisition; DIA, data-independent acquisition; nDIA, narrow-window DIA; LFQ, label-free quantification; IP2, Integrated Proteomics Pipeline; PASEF, parallel acquisition–serial fragmentation; FACS, fluorescence-activated cell sorting; iProChip, integrated proteomics chip; PiSPA, pick-up single-cell proteomic analysis; Mad-CASP, mass-adaptive coating-assisted single-cell proteomics; PCR, polymerase chain reaction; OAD, oil–air–droplet; μPAC, micropillar array-based column; FAIMS, field asymmetric ion mobility spectrometry; iPAD-1, integrated device for single cell analysis; nanoPOTS, nanodroplet processing in one pot for trace samples; SCoPE-MS, Single Cell ProtEomics by Mass Spectrometry; SCoPE2, Single-Cell ProtEomics 2; mPOP, Minimal ProteOmic sample Preparation.

Tissues are highly heterogeneous, comprising various cell types with distinct functions and environmental responses. Dissociating single cells from tissues results in the loss of spatial context, including cell location in tissues and extracellular matrix information. Spatial SCP can decipher how spatial context of cells influences their behavior, providing deeper insights into their biological processes and leading to, for example, better therapies and drug targets [47]. Laser capture microdissection (LCM) is widely employed in spatial proteomics. LCM precisely isolates a small group of target cells or even a single cell from solid tissues while preserving the tissues’ morphology and extracellular microenvironment. It is compatible with multiple tissue types, upstream preservation/staining methods, and downstream LC-MS analysis.

Spatial SCP workflows are more challenging and intricate. Tissues are first imaged, and then regions of interest (ROIs) are selected, isolated, and transferred to reaction vessels before a SCP workflow starts. For example, Mann’s group reported a Deep Visual Proteomics (DVP) technique, which combined imaging-based software, LCM, and LC-MS for single-cell spatial proteomics analysis [48]. DVP largely relies on an artificial intelligence-based image analysis software, Biology Image Analysis Software (BIAS), to execute deep learning-based cell segmentation and machine learning-based cell identification. After ROI selection, LCM and SCP workflows are carried out [48]. Other spatial SCP attempts include combining deep ultraviolet laser ablation (DUV-LA) or Zeiss PALM MicroBeam system with nanodroplet processing in one pot for trace samples (nanoPOTS) — a SCP workflow [49,50]. DVP and DUV-LA are not commercially available yet.

### Sample preparation

Proper sample preparation may be the most critical step in the MS-SCP workflow, as downstream analysis and overall data quality largely rely on its sensitivity, robustness, and reproducibility [51]. The design of the MS-SCP preparation workflow serves the same purpose of extracting as many protein molecules from cells as possible and delivering clean peptides into the LC-MS system. To achieve this goal, researchers eliminate the sample transfer and clean-up steps, minimize volumes, and adopt robotic operation whenever possible.

Generally, SCP sensitivity limitations stem primarily from sample loss via surface adsorption rather than instrument performance (Figure 1). Proteins and peptides are easily adsorbed to the surfaces of all kinds of materials [52]. A typical sample preparation workflow in bottom-up LC-MS analysis includes cell lysis, protein extraction, reduction, alkylation, digestion, and clean-up. Bulk samples are usually lysed by robust mechanical disruption (*e.g.*, sonication, bead beating, or homogenization) with obvious sample loss due to splashing and adherence. During multiple pipetting and tube transferring, proteins/peptides are also exposed to different surfaces, inevitably leading to sample loss via nonspecific adsorption [53]. While the overall sample loss and reproducibility are acceptable in bulk samples due to their large amount of starting materials, these factors may lead to significant changes and unreliable results in single-cell samples [9]. Wu et al. assessed the sample loss in a typical in-solution workflow with 500,000, 100,000, and 20,000 cells as starting materials and observed 15%, 72%, and 89% loss, respectively [54]. They reported only 76 proteins from 1000 processed cells but reported ∼ 2000 proteins from 0.1% of 1 × 10^6^ processed cells with the same workflow [54]. A single cell has far less protein content, which highlights the importance of preventing sample loss for a successful SCP analysis. Most MS-SCP sample preparation workflows are either completed in a single pot or are container-less without the need to transfer samples [55].

Clean-up procedures are required for bulk sample preparation to remove LC-MS-incompatible chemicals and precipitates before they are subjected to the LC-MS system [56,57]. The strategies usually incorporate more than one of the following protocols: protein precipitation and redissolution, buffer exchange, and desalting on C18 columns. Apparently, single-cell analysis cannot afford the risk of sample loss and subsequent sample variability. One option is to use trypsin and LC-MS-compatible surfactants, such as RapiGest (removable by acid treatment before LC-MS/MS acquisition) or N-dodecyl-β-D-maltoside (DDM; LC-MS-compatible at low concentrations) [58,59]. Another option is to skip detergents and unnecessary steps. Due to the trace material and interference, detergents or chaotropic agents are not necessary to facilitate cell lysis, protein solubilization, and denaturation of single cells. The minimal cellular debris, nucleotides, and salts would not need to be removed before LC-MS analysis. Even reduction and alkylation are disregarded in some MS-SCP workflows [24,60,61].

Another way to reduce sample loss is to reduce the sample preparation volume. The smaller the exposure surface area, the less the surface adsorption. Reducing the volume also increases the protein/peptide concentration, which improves sample–reagent reaction rates and introduces less disruptors (such as mass tags and trypsin) [60,62]. The sample preparation volume in MS-SCP ranges from a few microliters to nanoliters. The former usually offers more accessible options, while the latter, as a trade-off, may introduce reproducibility risk due to random errors in sample transfer and evaporation for such low volumes [4,43,63]. These issues can be largely mitigated by automatic workflows and artificial hydration [43] or protection by an oil layer [34].

Multiple innovative MS-SCP sample preparation methods have been reported in recent years (Table 2). In mass-adaptive coating-assisted single-cell proteomics (Mad-CASP), containers were coated with a designed hydrophobic peptide to prevent sample loss from protein binding or adhesion to container surfaces. A lysine was inserted into the peptide sequence after every four amino acids to generate 5-AA peptide fragments following trypsin digestion, so that these low-mass fragments could be excluded during MS data acquisition without interfering with single-cell peptide identification [33]. This hydrophobic peptide caused a 58% increase in protein numbers for single cells and had wide application from tubes to 384-well plates. This method could be adopted by any proteomic lab due to its simple operation and independence on special devices. nanoPOTS, a microfluidic sample preparation platform with fabricated nanowell chips, was one of the pioneers in MS-SCP [7,41,61]. This platform dramatically reduced the preparation volume to a few hundred nanoliters, thus minimizing the surface adsorption. The design of sealing a nanowell during incubation effectively reduced droplet evaporation. Similar nanodroplet-based devices were reported by others teams, such as OAD [34], nano-ProteOmic sample Preparation (nPOP) [64], and proteoCHIP [45]. Coupling these with the CellenONE single-cell sorting system further enhanced the nanodroplet-based approach’s performance and throughput, enabling parallel processing of up to 576 single cells per run [45]. The commercially available Rapid-Digestion Trypsin/Lys-C Kit simplifies the multi-hour and multi-step SCP preparation workflow to one-hour and one-step preparation. The high-temperature stabilized enzymes enable simultaneous cell lysis, protein denaturation, and digestion, achieving results similar to those of the conventional multi-step methods [32,39].

### LC separation

The sensitivity of LC-MS platforms is quite critical for overall data quality in MS-SCP. The factors that determine overall sensitivity of the platform include LC flow rate, LC resolution, and ionization efficiency (conversion of solution-phase molecules to gas-phase ions), as well as the MS instrument itself [65]. In the platform of nanoflow LC coupled with electrospray ionization MS (nanoLC-ESI-MS), the most common combination for bottom-up analysis, it has been proven that lower flow rates significantly enhance ionization efficiency and thus proteomic analysis sensitivity, as lower flow rates produce smaller electrospray droplets which cause less ion suppression [66–68]. Low flow rates also increase peptide ion concentration but decrease the solvent-derived contaminants in MS analysis [42].

To maintain LC separation resolution at lower flow rates, the internal diameter (i.d.) of the LC column has to be narrowed. Considering the balance of sensitivity, loading capacity, and robustness, the specifications of common nanoLC analytical columns are 75-µm i.d. and 1.7–5-µm particles, operated at a flow rate of 300 nl/min [69]. In the MS-SCP field (Table 2), a 30-µm-i.d. narrow-bore column operating at a flow rate of 60 nl/min was first reported in 2018 [41]. Although maintaining stable flow and particle packing were more difficult than in common columns, the team achieved good results by combining such narrow columns with their nanoPOTS sample preparation approach. The authors further developed an even narrower 20-µm-i.d. analytical column, which was utilized after a split flow column to achieve a 20 nl/min flow rate from 250 nl/min programmed nanoLC pump flow [42]. A 22-μm-i.d. narrow-bore column with an integrated ESI-tip and a split flow rate of 40 nl/min was also reported. The effects of 3-to-20-μm-i.d. ESI-tips were compared in this research, and the narrower ESI-tip presented better performance on signal intensities [40]. An alternative choice is the micropillar array-based column, termed μPAC. Nonporous particles have low binding capacities and thus reduce on-column losses, making μPAC columns well suited for ultra-low input samples such as single cells [70,71]. Additionally, the improved peak width and retention time (RT) robustness possibly benefit sensitivity [17]. Commercialized μPAC columns have been used in MS-SCP research [35,72]. A dual-column strategy was also proposed to improve throughput. While one column was running an effective LC gradient (peptide separation), the other column was in preparation for the next run by being washed and re-equilibrated [44].

As for the LC instrument itself, a commercial platform named Evosep One was launched in 2017. Evosep One claimed to be designed for robustness and high throughput, aiming for clinical and ultra-low input materials [73]. The platform has been successfully demonstrated to process 80 label-free single cells per day with a low flow rate of 100 nl/min and a short gradient of 15 min [27]. The continuous innovation and improvement of these LC columns and instruments undoubtedly promise a future in MS-SCP applications.

### MS data acquisition

Bottom-up LC-MS analyses can generally be categorized into two types according to data collection modes — data-dependent acquisition (DDA) and data-independent acquisition (DIA) — or based on protein quantification methods — label-free quantification (LFQ) and multiplexed quantification (Table 2). In DDA, peptide precursors are scanned and recorded at MS1, and the most abundant peptide precursors within a certain period are selected for fragmentation and sequencing. While DDA offers straightforward peptide identification and quantification, its major drawback for MS-SCP is the run-to-run reproducibility risk due to selection randomness, especially in such low peptide contents. In DIA, the entire target mass range is divided into sequential windows and systematically sampled. Theoretically, this approach enables the fragmentation and scan of all peptides. The identification and quantification of peptides and proteins should be more consistent across multiple acquisitions.

In the LFQ workflow, each single cell is acquired as an individual sample in either DDA or DIA mode. LFQ possess higher quantitative accuracy than multiplexed methods, more direct collaboration with automated workflows, and the potential to quantify nonpeptide molecules (*e.g.*, metabolites) in single cells [23]. Currently, the state-of-the-art LFQ method in DDA mode can detect over 2000 proteins from a single cell [39], while that in DIA mode reaches ∼ 5000 proteins per single cell [27]. A major challenge in LFQ is its limited throughput (12–80 single cells per day), with LC-MS runtimes ranging from 15 to 120 min [27,33]. The long data acquisition time makes it difficult for SCP research to match a similar scale as in single-cell transcriptomics. The dual-column strategy that spared sample loading, column washing, and equilibration time resulted in a throughput of 200 samples per day but at the expense of proteome depth [44].

In the multiplexed quantification DDA workflow, peptides derived from multiple single cells are labeled with unique isobaric tags [usually tandem mass tags (TMTs)] and pooled to generate one multiplexed sample of 9–16 single cells [28]. With the newly released 32-plex TMT sets, the labeling workflow produces much higher throughput than the LFQ workflow by simultaneously running up to 32 single-cell samples. The application of isobaric tags in MS-SCP was first introduced by the Single Cell ProtEomics by Mass Spectrometry (SCoPE-MS) approach in 2018 [28], in which the TMT-10plex protocol for 100 μg proteins per channel was revised to adapt for single cells and a carrier sample of 200 cells was included to boost peptide ion signals of single-cell samples in MS analysis. The higher-abundance peptides from the carrier provide more ions for the multiplexed sample and hence boost the precursor and fragment ions’ signals, leading to better sequencing quality for single-cell peptides [29]. The quantification bias possibly caused by carriers do cause concerns, and the proportion of carriers to single cells has been evaluated by several teams [74–76]. The suggested carrier proteome upper limit is 200× in multiplexed quantification.

The approach of carrier-assisted isobaric labeling quantification has been widely adopted in the MS-SCP field, reaching a quantification depth of 1000–1500 proteins per cell and a throughput of more than 250 cells per day [52,54,71]. While it matches LFQ in identification depth and surpasses LFQ in throughput, multiplexed quantification suffers from reporter-based quantification bias, more specifically, the ratio compression due to precursor interference, with which the apparent fold changes are less than the actual changes [77].

Considering the drawbacks of isobaric labeling in MS-SCP, researchers began to introduce the multiplexed concept into DIA to achieve both satisfactory throughput and quantitative quality. Traditional isobaric tags such as TMTs are not applicable in DIA. The MS2 spectra of DIA are already much more complicated than those in DDA. With the isobaric labeling approach, the complexity would be multiplied by the number of labeled samples and become too convoluted to identify peptides [78]. Researchers have turned to the multiplexed DIA strategy to seek a balance between throughput and quantification accuracy. They used only 2–3 heavy amino acid tags or nonisobaric chemical tags for each peptide to provide better quantification accuracy than TMT-DIA and higher throughput than LFQ-DIA [79,80]. In MS-SCP, a multiplexed DIA approach termed plexDIA has been reported, in which three single cells are labeled with triplex amine-reactive, nonisobaric isotopologous mass tags [mass differential tags for relative and absolute quantification (mTRAQ)] to be simultaneously analyzed. This approach reached a quantification depth of ∼ 1000 proteins per cell and a throughput of 144 cells per day, and exhibited fewer missing values than TMT-DDA [81]. Multiplexed DIA is in its infancy stage compared to TMT-DDA, considering the throughput and proteome depth. Nonetheless, multiplexed DIA may create a new and promising path in the MS-SCP field.

The Orbitrap and timsTOF series of MS have been reported with sufficient sensitivity for SCP and have been widely applied (Table 2). The Orbitrap series with higher resolution in DDA data acquisition mode have been historically dominant for protein identification and quantification in the proteomic field. In recent years, with the maturation of DIA data acquisition schemes and analysis software, as well as the development and rapid improvement of timsTOF MS, DIA strategies have dominated traditional methods in bulk proteomics and SCP analyses. Although timsTOF instruments provide lower-resolution mass spectra than Orbitrap MS, they are designed for low-input samples and exhibit high ion utilization efficiency under DIA parallel acquisition–serial fragmentation (PASEF) mode [82]. Ctortecka et al. identified 4000 proteins from a single cell using the 2023-released timsTOF Ultra MS [8]. The performance of the 2024-launched timsTOF Ultra 2 can be expected. Another exciting advance was the 2023-released Orbitrap Astral, with higher resolution and speed for MS1 scans acquired by the Orbitrap and MS2 scans acquired by the Astral in parallel. Ye et al. reported ∼ 5000 proteins from a single cell by using the narrow-window DIA (nDIA) method on the Orbitrap Astral [27]. This is the best proteome coverage in SCP analysis to date.

### Data analysis for MS-SCP

Following breakthroughs in MS-SCP sample preparation and data acquisition, corresponding data analysis methods have gradually emerged. Notably, changes in LC-MS parameters can affect algorithm performance, and there are fundamental differences between MS-SCP and MS-based bulk proteomics data [83]. The MS data analysis process usually involves four steps: peptide identification, peptide quantification, protein identification, and protein quantification. Current mainstream MS2-based peptide identification software, such as Proteome Discoverer (PD), MaxQuant, Spectonaut, and DIA-NN, was developed for bulk samples [84–87]. According to the characteristics of bulk proteomics data, these classic software/algorithms rely on the assumptions as follows: (1) sample ion intensities are generally higher than background noise, (2) spectra of MS-acquired fragment ions match the theoretical fragment ions, and (3) spectra of the same precursor ions from different samples exhibit high similarity [9]. However, in single-cell samples, low-abundance peptides derived from small protein contents can only provide limited fragment ions, which may distort the MS2 spectra [88]. The different characteristics and relatively lower signal-to-noise ratio in SCP spectra may affect the software’s judgment in the spectrum matching process [88].

Missing values are a considerable issue in the quantitative comparison of bulk samples and even more serious in single-cell studies with less quantifiable peptides/proteins and larger cohorts. The SCP field has employed a match-between-runs (MBR) algorithm in MaxQuant to reduce missing values [30,33,40,41]. Ions of low-input peptides may be acquired in MS1 spectra but not selected for MS2 identification [85]. MBR identifies these potential peptides by matching the accurate mass and aligned RT from another run [89].

A few other algorithms have been developed to decrease the number of missing values and increase proteome completeness in MS-SCP. Ion current extraction Re-quantification (IceR) adapts the concept of peptide identity propagation (similar to MBR) to enhance data integrity and quality for the LFQ-DDA approach [90]. Data-driven Alignment of Retention Times for IDentification (DART-ID) uses both ion RT and spectra to improve the characterization of weak-spectrum peptides. DART-ID can improve the number of data points by 30%–50% in bulk and single-cell samples [91]. The same team has also reported Data-driven Optimization of MS (DO-MS) which interactively visualizes LC-MS data from all levels, diagnoses potential issue, and provides rational optimization strategies [92].

Different SCP databases have also been recently reported. Single-cell Proteomic DataBase (SPDB) provides a large-scale integrated SCP database, including MS-based and antibody-based datasets, while being user-friendly and convenient [93]. SingPro contains MS-based and flow cytometry-based datasets with detailed experimental descriptions and expression profiles of the analyzed proteins [94].

## Biological applications of MS-SCP

**Figure 3:**
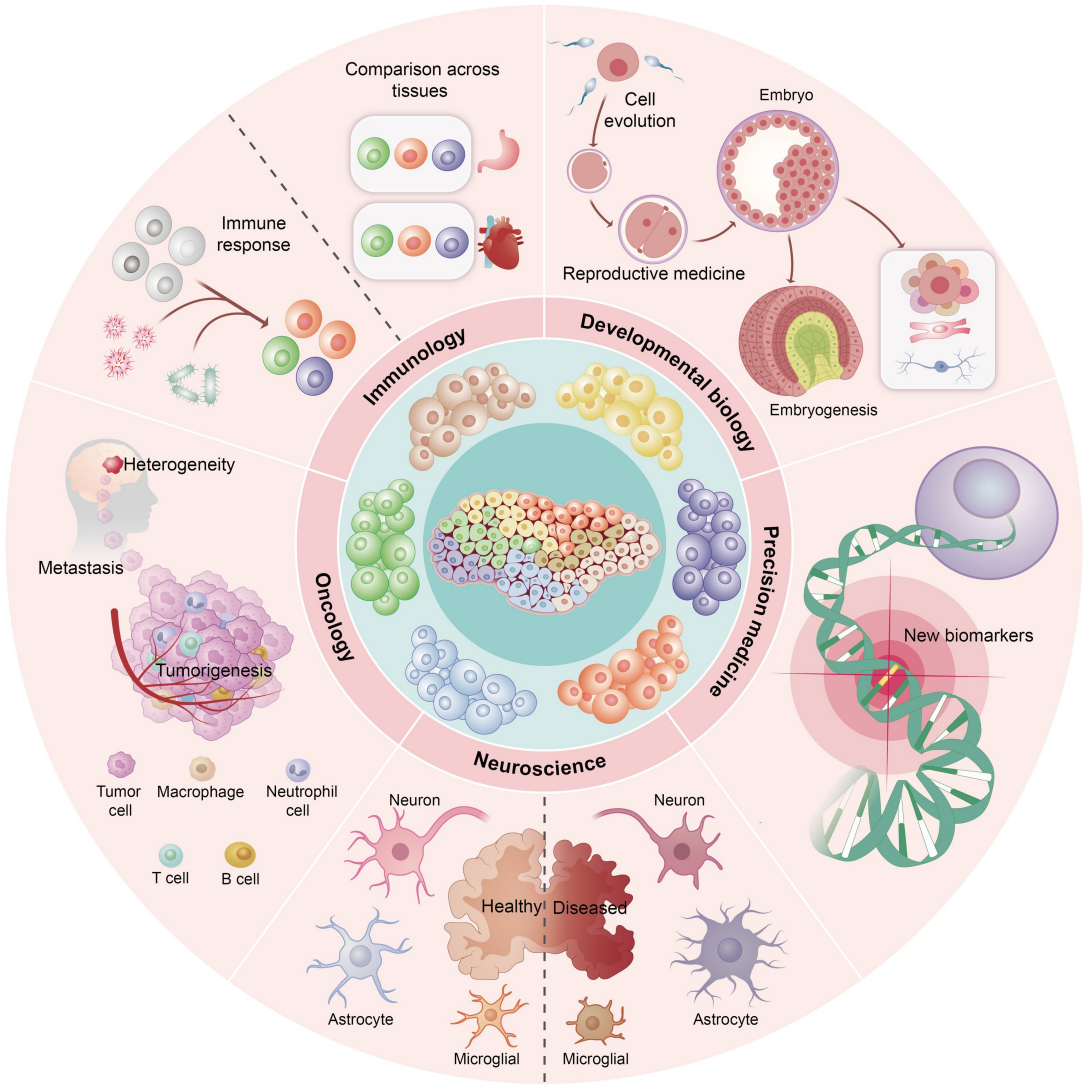
Applications of MS-SCP in key biomedical fields

MS-SCP has not only made significant progress in technology but also revolutionized the field of biology and medicine. Its ability to identify rare cell populations and understand cellular heterogeneity makes it a valuable tool for numerous applications, such as developmental biology, neuroscience, immunology, oncology, and other important basic research or clinical fields (**Figure 3**).

Li et al. performed MS-SCP analysis to circulating CD34^+^ cells from chronic total occlusion (CTO) patients, whose function remains unclear in disease progression due to the extremely limited cell population in peripheral blood [33]. The MS-SCP strategy successfully revealed novel subpopulations of CD34^+^ cells with different functions, as well as potential drug targets. These more precise subsets and their specific functions were only discovered at the single-cell level, but not at the multi-cell level.

In a study investigating islet cell heterogeneity using a high-fat diet (HFD)-induced mouse model, scRNA-seq was applied to identify proper surface markers of distinct subpopulations, and MS-SCP was then applied to the sorted cells to explore the functional status of each islet cell subset [95]. Combining different single-cell omics technologies, this team reported dynamic changes in both proportions and functions of islet cell subsets, presenting the adaptation of islets in response to high-fat stress. These new findings may provide novel ideas for diabetes treatment by enhancing islet function.

Some additional studies demonstrate the broad applicability of MS-SCP. For example, researchers profiled single motor neurons and interneurons from human spinal cord LCM sections, demonstrating the capability of distinguishing neuronal subpopulations at the single-cell level and identifying proteins related to cell type-specific functions under healthy or diseased conditions [30]. Mann’s group applied spatial SCP via single-cell DVP (scDVP) technology to murine liver, revealing the spatial heterogeneity of the cellular proteome in liver tissue [96]. The significant differences between portal and central zones in scDVP data were consistent with scRNA-seq data and FACS-based proteomic data [96]. With nanoliter-scale OAD chips, the proteome analysis of single human oocytes and blastomeres was able to be performed, enhancing the understanding and further exploration of functional protein networks during human preimplantation development [97]. Slavov’s group adopted the nPOP platform to quantify and interpret the covariation across single melanoma cells and found that the diverse cell populations were differentially involved in biological processes, such as cell division cycle and drug resistance [58]. Most recently, Woo et al. used CellenONE to isolate single prostate cancer cells with or without treatment of cisplatin and performed MS-SCP analysis on 144 single cells. They quantified the difference pattern in protein expression of drug-resistant cells and classified the cells into 3 subpopulations with differential pathway enrichment [98]. Collectively, these studies support the huge potential of MS-SCP in elucidating cellular functions, disease progress and therapy, drug resistance, and precision medicine.

## Perspectives of MS-SCP development and applications

SCP has great potential to revolutionize the field of biology and medicine (Figure 3). Its ability to identify rare cell populations and understand cellular heterogeneity makes it a valuable tool for numerous applications, *e.g.*, tumor heterogeneity and drug resistance studies, circulating tumor cell (CTC) analysis, and tumor immunity research. Tumor cells are continuously selected for more aggressive and drug-resistant offspring clones through spontaneous mutation during chemotherapy, thereby exhibiting a high degree of heterogeneity. Proteome analysis of tumor cells at the single-cell level can more accurately establish a relationship between gene mutation and protein expression, more comprehensively elucidate different drug resistance mechanisms, and help develop more potent anticancer drugs. CTC analysis, a recent breakthrough in precision medicine, is another important field to which SCP can contribute. SCP analysis of CTCs can provide critical protein data on the malignant degree of tumor tissue and the activation status of signaling pathways. For immune system and tumor immunity research, SCP analysis can deliver vital information about the biological roles of immune cells, which are highly differentiated in terms of cell types and functions. A comprehensive profiling of protein expression statuses in various types of immune cells is important for the design and development of the next-generation immunotherapy drugs.

MS-SCP has made significant progress in sample preparation, data acquisition, and data analysis; however, it is currently a young field. More efforts are needed to improve the technology and extend its applications. LFQ for SCP has become an efficient analysis approach, enabling the identification and quantification of over 5000 proteins in a single HeLa cell. Compared with the labeled SCP quantification approach, the throughput is a major challenge for the label-free method. Meanwhile, recently, the throughput has been significantly improved with the update of MS data acquisition technologies to enhance the MS sensitivity, resolution, and scan speed. The further development of algorithms and software tools with deep learning assistance would upgrade the utilization of acquisition data and improve the depth and coverage of SCP analysis. There is a big gap in throughput between MS-SCP and single-cell transcriptomics. A potential direction to further improve the throughput of MS-SCP is to revolutionize the labeled quantification approach, enabling more single cells to be analyzed and more proteins to be identified in a single MS data acquisition run in a shorter timeframe.

With high-throughput data acquisition and rapid data accumulation, SCP could become an indispensable technology for both academic research and medical applications.

## CRediT author statement

**Siqi Li:** Conceptualization, Investigation, Writing – original draft, Visualization. **Shuwei Li:** Supervision, Writing – review & editing. **Siqi Liu:** Supervision, Writing – review & editing. **Yan Ren:** Supervision, Conceptualization, Writing – review & editing. All authors have read and approved the final manuscript.

## Competing interests

Siqi Li and Siqi Liu are the current employees of BGI-Shenzhen. Shuwei Li is the current employee of Nanjing Apollomics Biotech Inc. All the other authors have declared no competing interests.
